# A coddling of the sagittal suture: inequality in spring-assisted expansion

**DOI:** 10.1007/s00381-024-06531-4

**Published:** 2024-08-02

**Authors:** Jinggang J. Ng, Ashley E. Chang, Dillan F. Villavisanis, Sameer Shakir, Benjamin B. Massenburg, Meagan Wu, Dominic J. Romeo, Jordan W. Swanson, Scott P. Bartlett, Jesse A. Taylor

**Affiliations:** Division of Plastic, Reconstructive and Oral Surgery, Children’s Hospital of Philadelphia, 3401 Civic Center Blvd, Philadelphia, PA 19104 USA

**Keywords:** Sagittal, Craniosynostosis, Outcomes, Spring, Cranial springs

## Abstract

**Purpose:**

We examined differences in long-term morphometric outcomes of spring-mediated cranioplasty (SMC) for various forms of isolated nonsyndromic sagittal craniosynostosis.

**Methods:**

A retrospective review was performed of children who underwent SMC from 2011 to 2020 at the Children’s Hospital of Philadelphia. Cephalic indices (CI), Whitaker grades, parietal bone thickness, and degree of suture fusion were assessed. Frontal bossing and vertex-nasion-opisthocranion (VNO) angles were compared to a normal control group.

**Results:**

Fifty-four subjects underwent surgery at age 3.6 ± 1.0 months with follow-up of 6.3 ± 1.8 years. Mean CI was 75.2 ± 4.1 at 5.9 ± 2.0 years postoperatively. Mean CI were 75.8 ± 4.1 (*n* = 32), 76.4 ± 4.0 (*n* = 22), and 77.1 ± 4.8 (*n* = 11) at 5, 7, and 9+ years postoperatively, respectively. Three (5.6%) required reoperation for persistent scaphocephalic cranial deformity. Fifty-one (94.4%) were Whitaker Grade I. On physical examination, 12 (22.2%) demonstrated craniofacial abnormalities. At long-term follow-up, there were no differences in frontal bossing angle (102.7 ± 5.2 degrees versus 100.7 ± 5.6 degrees, *p* = .052) and VNO angle (44.9 ± 3.3 degrees versus 43.9 ± 2.2 degrees, *p* = .063) between study and control groups. Younger age at surgery predicted a lower Whitaker grade, more normalized VNO angle, and greater change in CI during active expansion. Increased percentage fused of the posterior sagittal suture predicted a higher Whitaker grade, while decreased anterior fusion was associated with frontal bossing and temporal hollowing.

**Conclusions:**

Overall, children undergoing spring-mediated cranioplasty for sagittal craniosynostosis demonstrated maintenance of CI, favorable cosmetic outcomes, and a low reoperation rate at mid-term follow-up. Early intervention is associated with improved aesthetic outcomes, and regional fusion patterns may influence long-term craniofacial dysmorphology.

**Supplementary Information:**

The online version contains supplementary material available at 10.1007/s00381-024-06531-4.

## Introduction

Sagittal synostosis is the most common form of craniosynostosis [1, 2] with an incidence of 1 in 5000 births [3, 4]. It is characterized by a theme of scaphocephaly, with gradations of frontal bossing, occipital bulleting, and anterior shift of the cranial vertex [5–8]. The goals of surgical intervention are to correct deformational changes and remove growth constraints to normalize head shape and prevent ramifications of elevated intracranial pressure (ICP) [6, 9]. Historical treatment is a strip craniectomy or cranial vault remodeling (CVR) [9, 10]. While the former is less invasive, it is associated with high reoperation rates and less effective than CVR in normalizing cranial shape [6]. Over the years, several modifications have been made to augment this procedure, including postoperative molding orthosis, an extended strip craniectomy, and varying forms of lateral osteotomies. In 1986, Persing et al. reported on the use of indwelling springs to expand the skull in an animal model [11, 12]. Claus Lauritzen was the first to document the use of springs in humans [13], and this technique has since been adopted at many centers for the treatment of sagittal craniosynostosis [14–17].

Spring-mediated cranioplasty (SMC) offers several advantages over alternative techniques. In comparison to CVR, SMC is associated with decreased blood loss, shorter operative time, and reduced duration of hospitalization [16], even when accounting for the additional surgery required for spring removal [14, 18–20]. Studies suggest that this technique provides comparable correction of scaphocephaly to frontobiparietal remodeling [21] and other forms of subtotal CVR [18] at short-term follow-up. While endoscopic strip craniectomy with orthosis may also offer favorable outcomes with minimal morbidity [22–24], the postoperative helmeting requirement for 6 months or longer may impose a significant burden on patients and families [25, 26]. Our center adopted SMC in 2011, with early data demonstrating a favorable perioperative safety profile [27] and durability of scaphocephaly correction up to 5 years postoperatively [28]. However, the long-term outcomes remain largely unknown, with most studies limited to several years of follow-up in few patients [14, 16–18, 21, 28]. In keeping with our center’s track record of long-term outcomes reporting in craniosynostosis care [29, 30], we examined the functional, morphological, and surgical outcomes of subjects who underwent SMC with an eye toward differences in outcomes based on distinctive presenting characteristics. In other words, given the various presentations of sagittal craniosynostosis in infancy, are there variables that predict more favorable long-term results?

## Methods

With institutional review board approval, a retrospective review was performed of children who underwent SMC for isolated sagittal craniosynostosis from 2011 to 2020 at the Children’s Hospital of Philadelphia with at least four years of postoperative follow-up. Those with an associated syndromic diagnosis were excluded. Demographic data, medical history, computed tomography (CT) imaging, and clinical photographs were collected. Long-term postoperative cephalic indices (CI) were obtained within 12 months of the specified time point (i.e., CI at “5 years postoperatively” were measured at 5 years ± 12 months postoperatively). CI were measured on CT scans or by caliper on physical examination, using the formula:

$$\mathbf{C}\mathbf{e}\mathbf{p}\mathbf{h}\mathbf{a}\mathbf{l}\mathbf{i}\mathbf{c}\;\mathbf{I}\mathbf{n}\mathbf{d}\mathbf{e}\mathbf{x}(\mathbf{C}\mathbf{I})=\frac{\text{Biparietal Diameter}}{\text{Occipitofrontal Diameter}}\times 100$$

Whitaker grades were assigned on physical examination and/or review of most recent clinical photographs by an attending craniofacial surgeon using the schema [31] in Online Resource 1. Follow-up was defined as the period between spring insertion and the most recent clinical evaluation in our Department.

### Spring-mediated cranioplasty technique

The technique utilized at our center for SMC has been previously described [32]. We aim for surgical correction from 2.5 to 5 months of age depending on gestational age and phenotypic presentation. In severe cases of scaphocephaly, preoperative helmeting may be considered. After a 1–2 cm sagittal strip craniectomy, the surgeon selects 2 to 3 cranial springs varying in width, length, and force, based on patient age, bone thickness, and suture fusion characteristics [33–36]. Springs are generally inserted posterior to the coronal sutures and anterior to the lambdoid sutures. If a third spring is used, it is placed at the midpoint of the suturectomy, which typically corresponds to the cranial vertex. The removed suture is morselized and placed in the craniectomy site, deep to the springs, to facilitate osteogenesis [37]. Resorbable suture may be used to secure the springs to the surrounding periosteum. Springs are removed after 3 to 4 months as an outpatient procedure.

### Postoperative photogrammetric analysis

Standardized lateral photographs were imported to ImageJ (U.S. National Institutes of Health, Bethesda, Maryland, USA) and oriented to a Frankfort Horizontal plane. Frontal bossing was measured using the angle formed by the intersection of a horizontal plane through the nasion and a line traveling through the nasion tangential to the glabella [38–40]. The vertex, nasion, and opisthocranion (VNO) angle was measured using methodology described by Blum et al. [5] Age, sex, and race-matched photographs of patients with nonsyndromic cleft lip were used as a control group.

### Preoperative morphometric analysis

Among subjects with high-resolution preoperative CT imaging, we analyzed preoperative characteristics using established methodology [35, 41, 42]. Parietal bones were isolated on Mimics (Version 23.0, Materialise, Leuven, Belgium) and imported to 3-matic (Version 23.0, Materialise, Leuven, Belgium). Measurements were obtained using the “measure” tool. Percentage of suture fusion was calculated as the length of the fused suture divided by the total length of the suture or segments created by bisection into anterior and posterior halves or trisection into anterior, middle, and posterior thirds. The degree of suture fusion was categorized into binary variables of completely fused and partially or completely patent segments. Mean, median, and maximum parietal bone thickness were calculated. Suture thickness was measured at anterior, middle, and posterior points along the sagittal suture, located 1 cm posterior to the coronal suture, at the midpoint of the suture, and 1 cm anterior to the lambdoid suture, respectively.

### Statistical analysis

Descriptive statistics, Pearson correlation coefficient, Mann-Whitney *U* test, Student’s *t*-test, chi-squared test, and multivariate linear regression were used. Statistical analyses were performed on JASP (Version 0.18.1, JASP Team, 2023) with significance defined as *p* ˂ 0.05.

## Results

### Cohort characteristics

Demographic and perioperative data of 54 included subjects are displayed in Table 1. For 30 patients with high-resolution preoperative imaging, preoperative morphometric data are presented in Online Resource 2. Seven (13.0%) patients underwent preoperative helmet molding therapy for 1–2 months. Subjects underwent SMC at a mean age of 3.6 ± 1.0 months, with a mean expansion duration of 3.7 ± 0.6 months. The mean postoperative follow-up was 6.3 ± 1.8 years. Comorbidities included autism in 4 (7.4%) subjects and attention-deficit/hyperactivity disorder (ADHD) in 3 (5.6%).

**Table 1:** Cohort characteristics

Variable (*N* = 54)	*N* (%)
Sex	
Male	42 (77.8)
Female	12 (22.2)
Race	
White	44 (81.5)
Black or African American	3 (5.6)
Asian	1 (1.9)
Other	6 (11.1)
Comorbidities	
Autism	4 (7.4)
Attention-deficit/hyperactivity disorder	3 (5.6)
Perioperative details	
Preoperative helmeting	7 (13.0)
Age at spring insertion (months)	3.6 ± 1.0
Duration of spring expansion (days)	111.6 ± 18.2
Total spring force (Newtons)	25.0 ± 5.3
Postoperative helmeting	13 (24.1)
Postoperative follow-up (years)	6.3 ± 1.8

Continuous variables presented as mean ± standard deviation

### Aesthetic outcomes

**Fig. 1:**
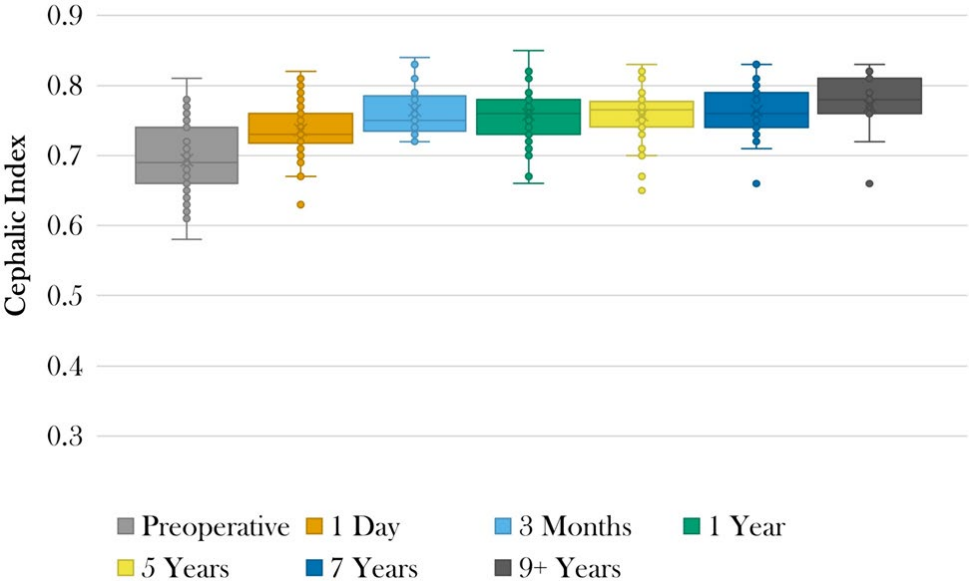
Box plot of cephalic indices preoperatively and at 1 day, 3 months, 1 year, 5 years, 7 years, and 9 years postoperatively

Mean preoperative CI was 69.3 ± 5.0 (*n* = 47) (Table 2). Mean CI was 75.2 ± 4.1 at 5.9 ± 2.0 years postoperatively. CI measured at standardized preoperative and postoperative time points are presented in Table 3 and Figure 1. Mean CI were 75.8 ± 4.1 (*n* = 32), 76.4 ± 4.0 (*n* = 22), and 77.1 ± 4.8 (*n* = 11) at 5, 7, and 9+ years postoperatively, respectively. Postoperative CI were greater than preoperative measurements at all time points (all *p* < .001).

**Table 2:** Cranial shape outcomes

Variable (*N* = 54)	*N* (%)
Cephalic index	
Preoperative measurement	69.3 ± 5.0
Most recent measurement	75.2 ± 4.1
Change in preoperative to most recent measurement	5.4 ± 4.5
Elapsed time from surgery to most recent measurement (years)	5.9 ± 2.0
Whitaker classification	
Grade I	51 (94.4)
Grade II	2 (3.7)
Grade III	1 (1.9)
Grade IV	0 (0.0)
Elapsed time from surgery to Whitaker grade (years)	5.5 ± 2.1
Physical examination findings	
Scaphocephaly	7 (13.0)
Temporal hollowing	5 (9.3)
Occipital bulleting	4 (7.4)
Frontal bossing	3 (5.6)
Elapsed time from surgery to most recent examination (years)	6.3 ± 1.8

^a^Continuous variables presented as mean ± standard deviation

**Table 3:** Cephalic index over time

Time point	*N*	CI	Percentage change from preoperative CI	*p*
Preoperatively	47	69.3 ± 5.0		
1 day postoperatively	54	73.6 ± 4.1	6.7 ± 5.9	< .001
3 months postoperatively	17	76.5 ± 3.6	10.4 ± 6.3	< .001
1 year postoperatively	35	75.9 ± 4.2	8.9 ± 7.2	< .001
5 years postoperatively	32	75.8 ± 4.1	8.1 ± 6.1	< .001
7 years postoperatively	22	76.4 ± 4.0	9.6 ± 6.0	< .001
9 years+ postoperatively	11	77.1 ± 4.8	10.2 ± 6.4	< .001

*CI*, cephalic index

Data presented as mean ± standard deviation

Among the cohort, 51 (94.4%) were classified as Whitaker grades I and 3 (5.6%) as grade II at 5.5 ± 2.1 years postoperatively. No subjects were graded as grade III or IV. On physical examination at 6.3 ± 1.8 years postoperatively, 12 (22.2%) had any craniofacial abnormalities. Seven (13.0%) demonstrated scaphocephaly, 5 (9.3%) had temporal hollowing, 4 (7.4%) had occipital bulleting, and 3 (5.6%) had frontal bossing on most recent physical examination. There were no significant differences in frontal bossing angle (102.7 ± 5.2 degrees versus 100.7 ± 5.6 degrees, *p* = .052) and VNO angle (44.9 ± 3.3 degrees versus 43.9 ± 2.2 degrees, *p* = .063) between study and control groups at a mean age of 6.2 ± 2.0 (Table 4). Age at photograph was inversely correlated with frontal bossing angle in the combined cohort (*r*(106) = *−* 0.280, *p =* .003) and study cohort (*r*(52) = *−* 0.326, *p* = .016), but not the control cohort (*r*(52) =* −*0.255, *p =* .063).

**Table 4:** Photogrammetric comparison

	Sagittal synostosis	Control group	*p*
*N*	54	54	
Age at photograph (years)	6.2 ± 2.0	6.1 ± 2.0	.848
Frontal bossing angle (degrees)	102.7 ± 5.2	100.7 ± 5.6	.052
VNO angle (degrees)	44.9 ± 3.3	43.9 ± 2.2	.063

*VNO*, Vertex-Nasion-Opisthocranion

Values presented as mean ± standard deviation

### Age at surgery

Younger age at surgery predicted a greater increase in CI during active expansion (preoperative to 3 months postoperatively) (*β* = − 0.283, *p =* .006) independent of preoperative CI, median parietal bone thickness, percentage fused of the suture, and total spring force. Moreover, older age at surgery predicted a higher Whitaker grade (*β* = 1.702, *p* < .001) and a greater VNO angle (*β* = 17.259, *p* = .014) independent of preoperative CI, parietal bone thickness, degree of suture fusion, and total spring force.

### Suture characteristics and outcomes

Increased percentage fused of the posterior half of the suture predicted less change in CI during active expansion (*β* = − 0.129, *p =* .019) independent of age at surgery, posterior spring force, median thickness, preoperative CI, and percentage fused of the anterior half. Moreover, increased percentage fused of the posterior third (*β* = 0.261, *p* = .002) and decreased percentage fused of the middle third (*β* = − 0.401, *p* = .002) predicted a higher Whitaker grade independent of age at surgery, preoperative CI, percentage fused of the other thirds, and total spring force.

Subjects with any craniofacial abnormalities on most recent physical examination were less likely to have complete fusion of the anterior half of the suture (44.4% versus 85.7%, *p* = .019). Children with frontal bossing (38.7% ± 53.1% versus 89.3% ± 26.3%, *p* = .008) or temporal hollowing (39.6% ± 53.2% versus 89.2% ± 26.5%, *p* = .009) on most recent physical examination had decreased preoperative percentage fused of the anterior third. Subjects with occipital bulleting on most recent physical examination were more likely to have decreased preoperative anterior suture thickness (0.8 ± 0.4 mm versus 1.6 ± 0.5 mm, *p* = .012). Patients with symptoms of headaches, head banging, and/or body tremors at most recent follow-up had decreased preoperative middle suture thickness (2.1 ± 0.5 mm versus 3.1 ± 0.8 mm, *p* = .026).

### Other outcome predictors

Subjects with occipital bulleting on most recent physical examination had decreased posterior spring force (8.1 ± 1.4 versus 9.8 ± 1.6 N,* p* = .045), while those with scaphocephaly had decreased middle spring force (8.2 ± 1.4 versus 9.7 ± 1.5 N, *p* = .033). Lower preoperative CI was predictive of a lower most recent CI (*β* = 0.523, *p* = .005) independent of age at surgery, total spring force, median thickness, and degree of suture fusion.

Children who underwent additional surgery had a lower CI at 1 year postoperatively (69.0 ± 2.6 versus 76.5 ± 3.7, *p* = .002) and decreased total change in CI (preoperative to most recent measurement) (− 0.7 ± 5.1 versus 5.9 ± 4.2, *p* = 0.013). Those with temporal hollowing had a lower immediate postoperative CI (66.4 ± 6.8 versus 69.7 ± 4.7, *p* = .002), and lower CI at 1 year (69.5 ± 3.5 versus 76.3 ± 4.0, *p* = .025), 5 years (69.0 ± 5.3 versus 76.5 ± 3.3, *p* = .001), and 7 years postoperatively (68.5 ± 3.5 versus 77.1 ± 3.1, *p* = .001). Subjects with occipital bulleting had a lower preoperative CI (62.5 ± 3.7 versus 70.0 ± 4.6, *p* = .003), as well as lower CI at one day (68.5 ± 4.4 versus 74.1 ± 3.8, *p* = .008) and 7 years postoperatively (68.5 ± 3.5 versus 77.1 ± 3.1, *p* = .001).

Patients with scaphocephaly on most recent physical examination had a lower CI preoperatively (65.2 ± 4.6 versus 70.0 ± 4.8, *p* = .026), immediately postoperatively (70.1 ± 3.1 versus 74.2 ± 4.0, *p* = .014), 3 months (72.7 ± 1.2 versus 74.2 ± 4.0, *p* = .041), 1 year (72.0 ± 4.1 versus 76.4 ± 4.0, *p* = .048), 5 years (66.0 ± 1.4 versus 76.4 ± 3.2, *p* < 0.001), and 7 years postoperatively (71.7 ± 6.0 versus 77.1 ± 3.2, *p* = 0.023). History of preoperative or postoperative helmeting was not associated with the presence of abnormalities on physical examination, most recent CI, frontal bossing angle, or VNO angle (all *p* > 0.05).

### Complications

Perioperatively, 5 (9.3%) required transfusion, 1 (1.9%) had a hematoma, 1 (1.9%) developed a nonoperative seroma, and 1 (1.9%) experienced spring dislodgement requiring reoperation (Table 5). Our team generally implements a hemoglobin threshold of 7.0 g/dL for transfusion, particularly if the patient is symptomatic. Three (5.6%) patients required subsequent cranial vault surgery. These subjects underwent posterior vault reconstruction at 1.6, 2.1, and 4.7 years following initial surgery for cranial dysmorphology. Of these 3 patients, one subsequently underwent fronto-orbital advancement due to bitemporal narrowing, persistent anterior scaphocephaly, and worsening headaches. At most recent follow-up, 11 (19.6%) patients among the cohort reported intermittent headaches, and 2 (3.6%) reported body tremors and/or head banging. Subsequent work-up—which routinely includes neuro-ophthalmology referral, optical coherence tomography (OCT), and CT imaging—did not demonstrate clear evidence of elevated ICP. For both patients with intermittent episodes of body tremors and/or head banging, these symptoms were considered to be related to potential seizure activity rather than increased ICP on neurological evaluation. None demonstrated papilledema on fundoscopic examination at 4.3 ± 2.8 years postoperatively (*n* = 50).

**Table 5:** Perioperative and postoperative complications

Complications (*N* = 54)	*N* (%)
Perioperative complications	
Transfusion requirement	5 (9.3)
Seroma	1 (1.9)
Hematoma	1 (1.9)
Spring dislodgement	1 (1.9)
Symptoms at most recent follow-up	
Headaches	11 (20.4)
Body tremors/head banging	2 (3.7)
Papilledema	0 (0.0)
Subsequent surgery	
Posterior vault reconstruction	3 (5.6)
Fronto-orbital advancement	1 (1.9)

## Discussion

This study characterized the outcomes of children who underwent SMC for sagittal craniosynostosis and revealed several key findings. First, correction of scaphocephaly was durable at long-term follow-up, and in a subset of the cohort, up to 9 years postoperatively. Second, most subjects had resolution of craniofacial dysmorphology both by clinical evaluation and in photogrammetric analyses. Third, SMC is associated with a favorable perioperative safety profile and low reoperation rate comparable to commonly performed alternatives [43–45]. Moreover, younger age at surgery predicted improved aesthetic outcomes. Anterior suture patency was associated with more severe frontofacial dysmorphology, while increased posterior fusion predicted less improvement in CI in the active expansion period and a worse Whitaker grade long-term. Children with the most severe scaphocephaly preoperatively remained more scaphocephalic long-term and those with less severe scaphocephaly tended to have the best morphometric outcomes. In other words, nonsyndromic sagittal craniosynostosis presents with variable suture fusion which may alter morphology and variably affect postoperative results. Our reporting of results in a more nuanced way may aid in surgeons selecting one operation over another based on preoperative differences.

**Fig. 2:**
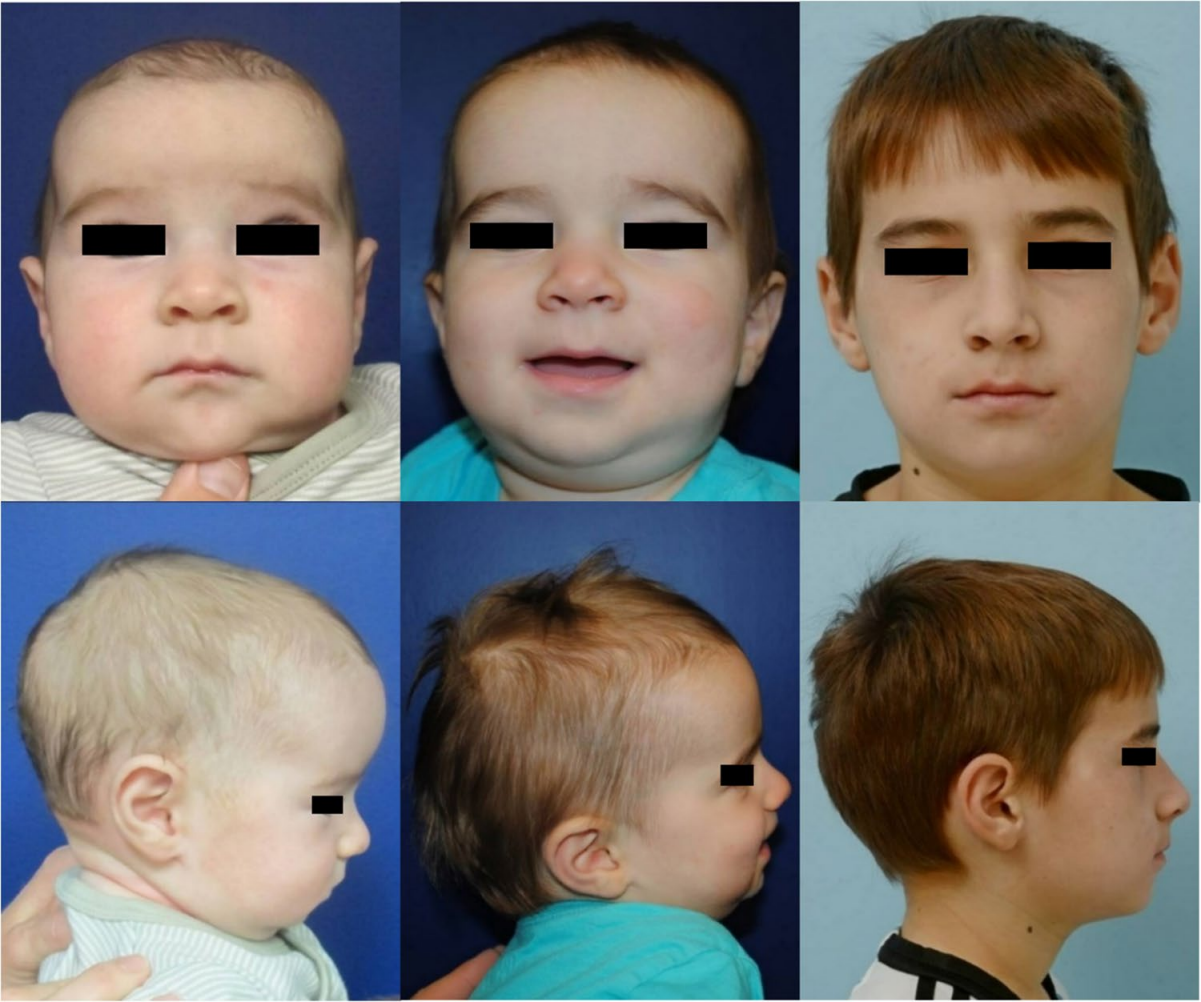
Frontal (top row) and lateral (bottom row) photographs of a patient at ages 3 months prior to surgical intervention, 1 year following spring-mediated expansion, and 9 years (left to right)

**Fig. 3:**
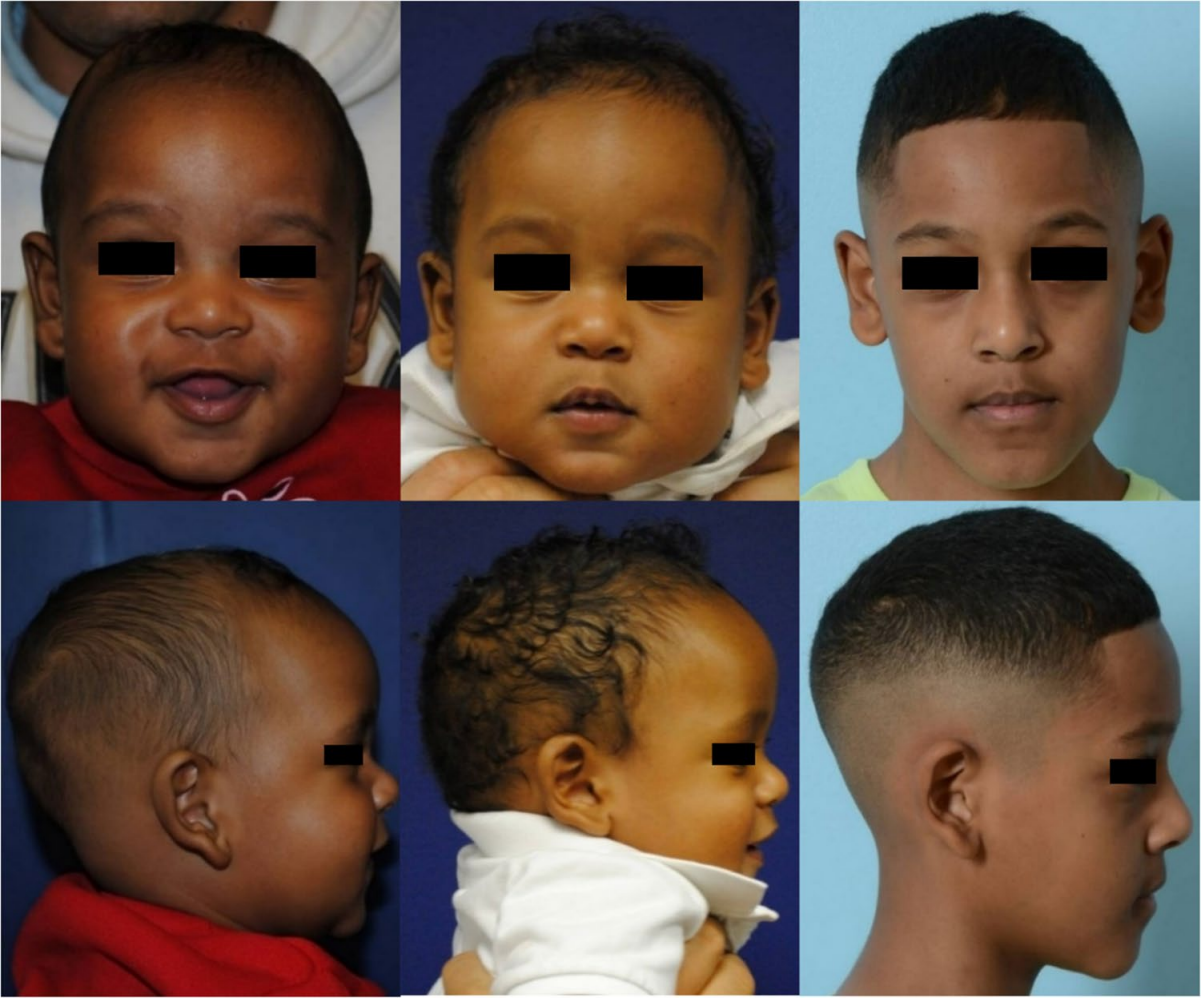
Frontal (top row) and lateral (bottom row) photographs of a patient at ages 4 months following spring insertion, 8 months following spring removal, and 9 years (left to right)

Among our cohort, CI demonstrated the greatest increase in the immediate postoperative and active expansion period and reached a plateau at 3 months postoperatively. In the subgroup of patients with over 9 years of postoperative follow-up, CI remained stable at the middle range of normal. To our knowledge, other studies have not reported on the cranial shape outcomes of SMC beyond 5 years postoperatively [18, 28]. Our study provides evidence to support the durability of scaphocephaly correction at long-term follow-up (Figures 2, 3 and Online Resource 3-4) in addition to reporting on some subtleties of cranial shape relating to fusion patterns of the sagittal suture.

Most of the cohort had favorable cranial shape outcomes at long-term follow-up with almost 95% classified as Whitaker grade I. Given the subjectivity of this classification system [46], we employed other tools to evaluate cranial shape including assessment of craniofacial abnormalities on physical examination and photogrammetric comparisons. Approximately one-fifth of the cohort demonstrated any craniofacial dysmorphology on physical examination, with the most common residual abnormality being mild scaphocephaly, followed by temporal hollowing. Photogrammetric analysis revealed no significant differences in frontal bossing and VNO angles between study and control groups at age 6. While frontal bossing angle trended toward being greater in the sagittal synostosis cohort, potential differences may diminish over time [7, 8, 47] as frontal bossing angle was inversely correlated with age in the study cohort. These assessments collectively imply that SMC provides adequate mid-term correction of craniofacial dysmorphology. As we have a growing cohort of patients reaching cranial maturity, we look forward to reporting on their outcomes in the coming decade.

In our cohort, less than 6% required subsequent vault expansion, primarily for cosmetic concerns. This number is similar to the reoperation rate reported in an early study from our center with a median follow-up of 1.5 years [28]. Our findings augment existing literature by providing a longer-term view on the durability of SMC with a mean follow-up of 6 years, and a subset of patients having reached 10 years of postoperative follow-up. Runyan et al. previously reported that 2 (1.1%) of 175 subjects undergoing SMC required revision surgery; however, follow-up was not reported, and subsequent procedures may not have been captured [18]. Notably, approximately one-quarter of our cohort reported symptoms of intermittent headaches, head banging, and/or body tremors at most recent follow-up, and a tenth were diagnosed with autism or ADHD. However, subsequent work-up did not reveal clear signs of elevated ICP and it was felt that further surgical intervention would not alter clinical course. While Runyan et al. found that 10% of patients who underwent SMC had headaches (in comparison to 28% in the CVR cohort), comparison with our data is challenging due to unclear follow-up [18]. As noninvasive methods of detecting intracranial hypertension are imperfect [48], we strive to monitor patients annually in a multidisciplinary craniofacial clinic with specialists from neuro-ophthalmology, neurosurgery, psychology, craniofacial surgery, and other disciplines. Overall, our study suggests that SMC is associated with a reasonable complication profile and low reoperation rate.

Importantly, our findings indicate that younger age at surgery predicts a lower Whitaker grade, more normalized VNO angle, and greater change in CI during the active expansion period. The age-dependent change in CI has been previously reported [28] and likely results from increased pliability of young infant calvaria [32]. However, the discriminatory effect of age on long-term cranial shape outcomes provides new insight into the importance of early intervention beyond its potential benefits to neurocognition [49, 50] and perioperative safety [51]. With mounting evidence to support early surgery, it is important to streamline care [52] for patients presenting at an older age to optimize outcomes and reduce downstream effects of sociodemographic disparities [53]. Of note, subjects with occipital bulleting in our cohort had decreased posterior spring force, while those with scaphocephaly had decreased middle spring force. These two findings may suggest a degree of undercorrection, and perhaps adjustment of regional spring forces may improve cosmetic outcomes.

Moreover, preoperative morphometric data revealed interesting insights into craniofacial dysmorphology. An increased percentage of posterior suture fusion predicted a higher Whitaker grade, while decreased fusion of the anterior segment was associated with the presence of frontal bossing and temporal hollowing. While the pathophysiologic forces underlying these patterns were not elucidated in our study, we hypothesize that anterior patency may reflect overgrowth or compensatory growth due to restriction in other dimensions [42]. Similar to reported findings in children undergoing CVR [7], those who were more scaphocephalic preoperatively remained more scaphocephalic long term. Our data support that fusion characteristics play a key role in craniofacial phenotype, and we hope to bring additional clarity to this point with future study. [41, 42].

We additionally found that preoperative middle suture thinness was associated with intermittent headaches, head banging, and/or body tremors at most recent follow-up. Previous studies have reported a link between calvarial thinness and intracranial hypertension [54, 55], as well as the preferential thinning of the posteromedial portion of the parietal bones [56]. The presence of symptoms in these patients at long-term follow-up may suggest some degree of persistent cephalocranial disproportion, though it is unclear whether these symptoms warrant additional vault expansion. It also emphasizes the primary point of this study—not all sagittal synostosis presentations are equal, and the nuances of presentation may predict the need for nuanced treatment differences.

Several limitations must be considered. This is a retrospective study and conclusion of causality is not possible. The measurement of frontal bossing and VNO angles from clinical photographs may be imprecise, particularly in patients with hair that obscured the curvature of the skull. Physical examinations were not performed in a protocolized manner; however, findings were recorded by an attending craniofacial surgeon using standard clinical descriptors. Finally, as this was a single-center investigation, it is unclear how our approach may generalize to other settings.

## Conclusions

Not all sagittal synostosis presentations are equal, and the nuances of presentation may predict the need for nuanced treatment differences. Spring-mediated cranioplasty for sagittal craniosynostosis is associated with maintenance of the cephalic index, favorable cosmetic outcomes, and a low reoperation rate at up to 10-year follow-up. Early intervention is associated with improved aesthetic outcomes, and regional fusion patterns may influence craniofacial dysmorphology. Future study is needed to guide differences in treatment based on preoperative phenotype.

## Supplementary Information

Below is the link to the electronic supplementary material.

Supplementary file1 (DOCX 14 KB) Online Resource 1. Whitaker Classification System

Supplementary file2 (DOCX 15 KB) Online Resource 2. Preoperative Morphometric Data

Supplementary file3 (DOCX 1323 KB) Online Resource 3. Oblique (left), lateral (middle), and vertex (right) views of 3-dimensional reconstruction of preoperative computed tomography (CT) scan of patient represented in Figure 2

Supplementary file4 (DOCX 472 KB) Online Resource 4. Frontal (left) and lateral (right) x-rays of patient represented in Figure 3 following spring-mediated expansion and prior to cranial spring removal
