# Supplementary material for: A coddling of the sagittal suture: inequality in spring-assisted expansion
Source: Childs Nerv Syst. 2024 Aug 2;40(12):3993–4002. doi: 10.1007/s00381-024-06531-4 (PMC11579197; doi:10.1007/s00381-024-06531-4)
Supplement: Supplementary file 1 — Supplementary file1 (DOCX 14 KB) Online Resource 1. Whitaker Classification System [file 381_2024_6531_MOESM1_ESM.docx]

**Supplemental Digital Content 1, Table.** Whitaker Classification System

| **Grade** | **Description** |
| --- | --- |
| I | No refinements or surgical revisions considered advisable. |
| II | Soft tissue or lesser bone contouring procedures considered advisable. |
| III | Major alternative osteotomies or bone grafting procedures necessary. |
| IV | Major craniofacial surgery necessary, duplicating or exceeding the original surgery. |
