# Supplementary material for: A coddling of the sagittal suture: inequality in spring-assisted expansion
Source: Childs Nerv Syst. 2024 Aug 2;40(12):3993–4002. doi: 10.1007/s00381-024-06531-4 (PMC11579197; doi:10.1007/s00381-024-06531-4)
Supplement: Supplementary file 2 — Supplementary file2 (DOCX 15 KB) Online Resource 2. Preoperative Morphometric Data [file 381_2024_6531_MOESM2_ESM.docx]

**Supplemental Digital Content 2, Table**. Preoperative Morphometric Data^a^

| **Variable** | **N = 30** |
| --- | --- |
| Sagittal Suture Fusion |  |
| Total Fused (%) | 90.4 ± 16.6 |
| Anterior Half Fused (%) | 89.4 ± 22.1 |
| Posterior Half Fused (%) | 91.4 ± 22.9 |
| Anterior Third Fused (%) | 84.3 ± 32.4 |
| Middle Third Fused (%) | 92.2 ± 24.3 |
| Posterior Third Fused (%) | 89.0 ± 27.2 |
| Calvarial Thickness |  |
| Parietal Bone (mm) | 1.7 (1.3-2.9) |
| Anterior Suture (mm) | 1.5 ± 0.5 |
| Middle Suture (mm) | 2.9 ± 0.9 |
| Posterior Suture (mm) | 2.6 ± 0.8 |
| ^a^Data presented as mean ± standard deviation or median (range). | |
