# Supplementary material for: A coddling of the sagittal suture: inequality in spring-assisted expansion
Source: Childs Nerv Syst. 2024 Aug 2;40(12):3993–4002. doi: 10.1007/s00381-024-06531-4 (PMC11579197; doi:10.1007/s00381-024-06531-4)

**Supplemental Digital Content 3, Figure.** Oblique (left), lateral (middle), and vertex (right) views of 3-dimensional reconstruction of preoperative computed tomography (CT) scan of patient represented in Figure 2.


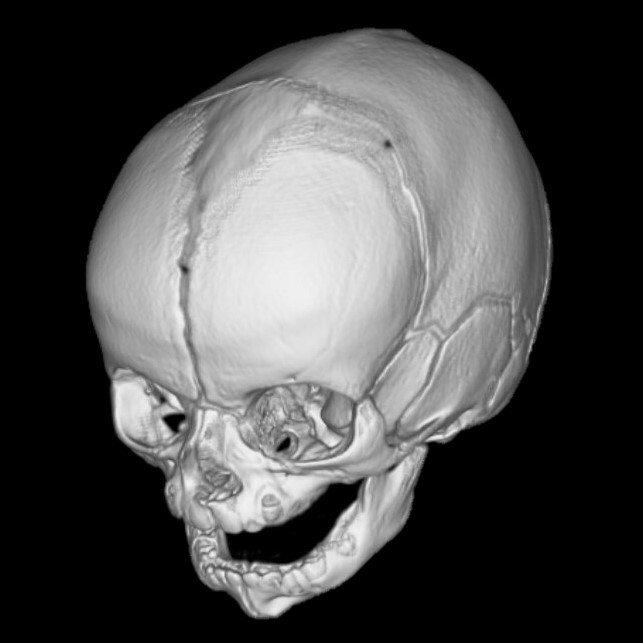

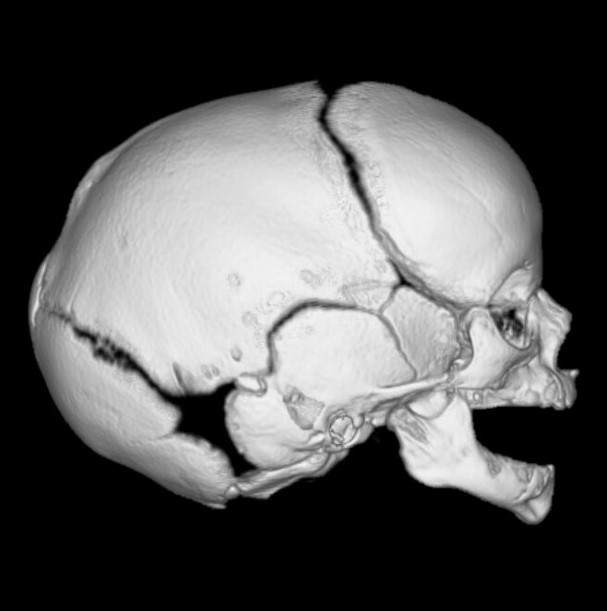

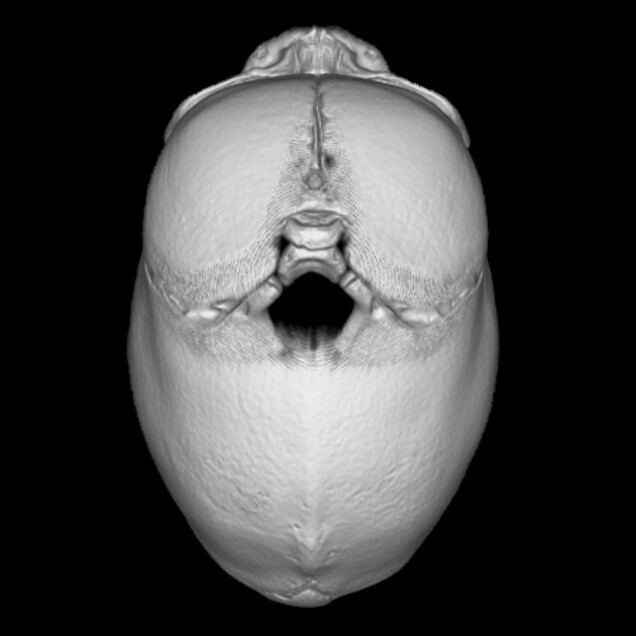


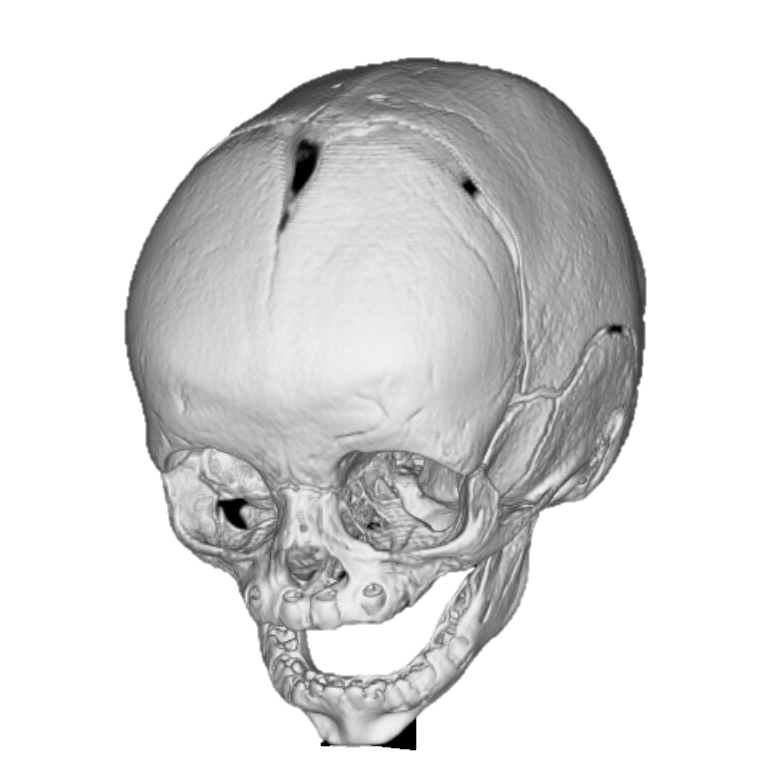

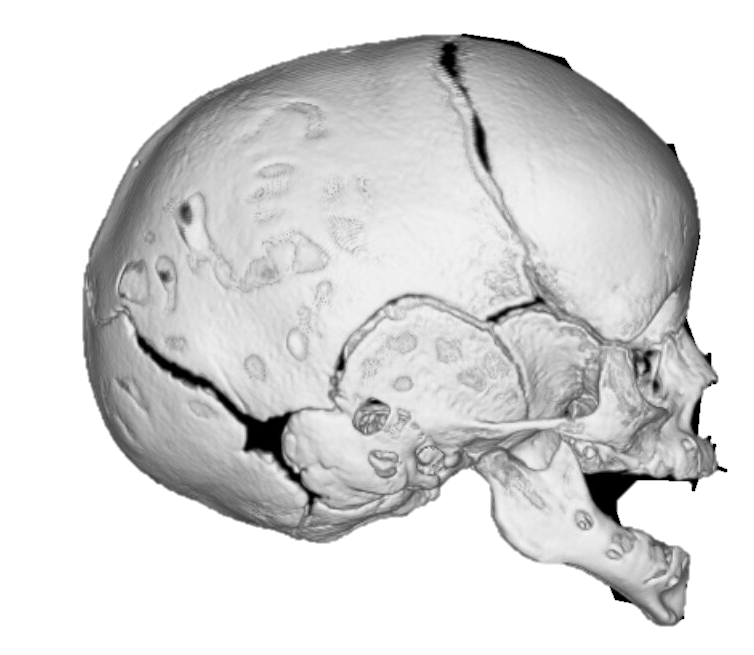


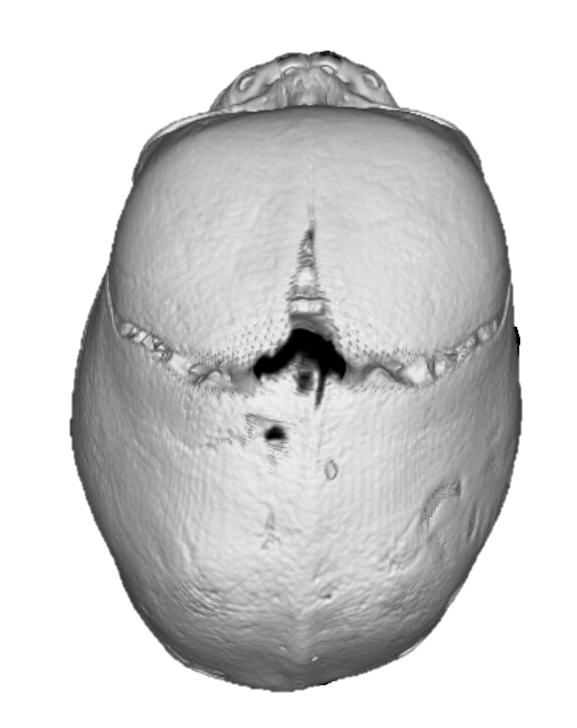

Supplement: Supplementary file 3 — Supplementary file3 (DOCX 1323 KB) Online Resource 3. Oblique (left), lateral (middle), and vertex (right) views of 3-dimensional reconstruction of preoperative computed tomography (CT) scan of patient represented in Figure 2 [file 381_2024_6531_MOESM3_ESM.docx]
