# Supplementary material for: A coddling of the sagittal suture: inequality in spring-assisted expansion
Source: Childs Nerv Syst. 2024 Aug 2;40(12):3993–4002. doi: 10.1007/s00381-024-06531-4 (PMC11579197; doi:10.1007/s00381-024-06531-4)

**Supplemental Digital Content 4, Figure.** Frontal (left) and lateral (right) x-rays of patient represented in Figure 3 following spring-mediated expansion and prior to cranial spring removal.


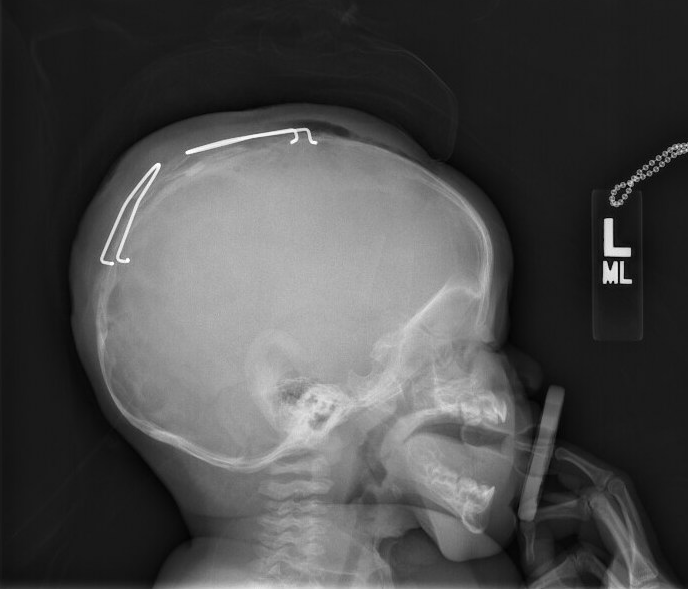

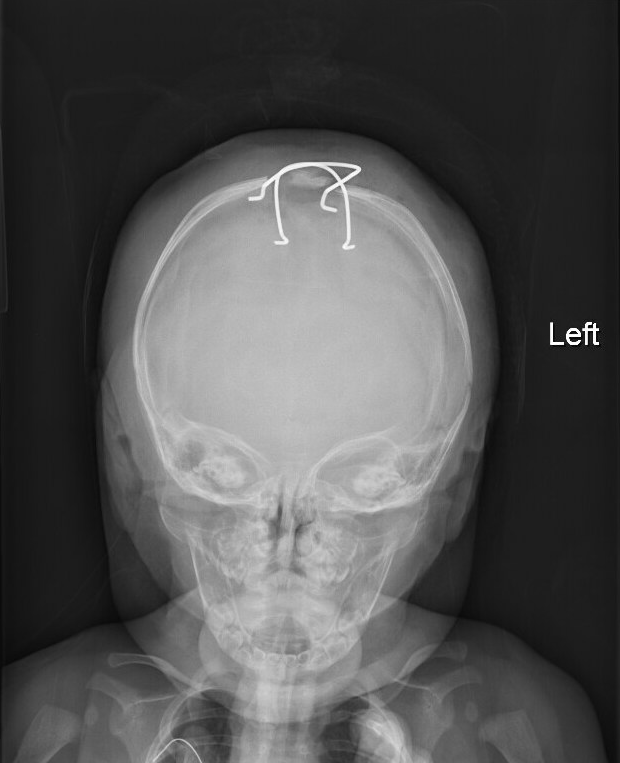

Supplement: Supplementary file 4 — Supplementary file4 (DOCX 472 KB) Online Resource 4. Frontal (left) and lateral (right) x-rays of patient represented in Figure 3 following spring-mediated expansion and prior to cranial spring removal [file 381_2024_6531_MOESM4_ESM.docx]
